# Dataset of physical features from natural water-bodies in a Mediterranean high-mountain context (Sierra Nevada, SE Spain)

**DOI:** 10.1016/j.dib.2022.107934

**Published:** 2022-02-10

**Authors:** J.L. Diaz-Hernandez, A.J. Herrera-Martinez

**Affiliations:** a IFAPA Camino de Purchil, Área de Recursos Naturales, Consejería de Agricultura, Pesca y Medio Ambiente, Junta de Andalucía, Granada 18080, Spain; b Espacio Natural Sierra Nevada (ESN), Área de Uso Público, Consejería de Medio Ambiente y Ordenación del Territorio, Junta de Andalucía, Pinos Genil, Granada 18191, Spain

**Keywords:** Cryo-oromediterranean water-bodies, Cryo-oromediterranean green fringes, Glaciology of Mediterranean mountains, Hillside instability, Landslides, Overdeepening, Thawing hydrology

## Abstract

This dataset was obtained over repeated field-trips to the Sierra Nevada Massif and contains the physical parameters of its recognised water-bodies. It therefore defines the general cartography of the area, with data on individual features regarding the geographical coordinates (x, y, z), dimensions (length, width, depth), flooded surface area, stored water volume, shoreline length, as well as the area of associated green fringes and the length of their borders. These data were basically obtained using straightforward techniques, such as GPS, tape measurements and photographic interpretation. The data were then previously used to define the role of these water-bodies in the hydrology of the massif: relationships between number of water-bodies and water volumes between 2700 and 3200 m a.s.l. regarding watersheds (Mediterranean, Atlantic and total massif), relationships with green fringes, moment of maximum snowmelt discharge and the estimation of different components of water volumes discharged during the main period of thaw. The formation patterns of each water-body were also identified in their situational context, and the role played by each formation process on the stored water volume: the water-bodies close to the peak line (2918 m mean altitude) are highly dependant on the glacial processes that created the hollows in which they are located. Slope instability created water-bodies mainly located at lower altitudes and are more fragile due intense slope dynamics. In any case, these hydrological data show a paradoxical behaviour because despite its higher xericity, the Mediterranean watershed generally has higher water contents than the Atlantic. The cause of this hydrological imbalance between watersheds seems to be unrelated to the formation processes of the existing water-bodies.

Sierra Nevada is considered to be clearly representative of a high mountain Mediterranean environment, where the data collected are a starting point to define the different habitats or for investigation of the hydrological processes of the massif and their evolution. Lack of such data is often a problem that in the present case is solved with this contribution.

## Specifications Table

**Table:** 

Subject	Environmental Sciences
Specific subject area	Nature and landscape conservation, Hydrology of water-bodies, Earth-surface processes.
Type of data	Table, expressed as a database in Excel format. Chart including all the inventoried waterbodies, identified by an order number. Figures, for clarifying some specific aspects.
How the data were acquired	The inventory was carried out during visits to each water-body over a long observation period (approx. 18 years), given the difficulty of access through snowfall. Geographical coordinates were obtained using a portable GPS (Garmin eTrex 10) and “Google Earth” and “Iberpix” visors (Instituto Geográfico Nacional, IGN) [1,2] (in lab), as well as the specific features of each formation pattern. Dimensions were measured using metric tape, and water depth was determined using a single seat rubber boat. The cartography of water surfaces and related green fringes is based on orthogonal aerial photographs (detailed images downloaded from Google Earth Pro 7.3.4.8248 (64-bit), Landsat 8) [1]. These were contrasted with field data, measurements performed using Canvas X Pro software. The database was made as an Excel sheet, where the volumes were determined.
Data format	Raw: UTM coordinates, dimensions (length, width and depth) and qualitative formation patterns. Analysed: water surface areas and associated shorelines, green fringe surface areas and associated perimeters. Filtered: volumes after defining categories. All measures were expressed in the International System of Units (SI).
Description of data collection	This inventory describes all the natural water-bodies preferably ≥ 2700 m above sea level (a.s.l.). Three cases were exceptions as indicated in [3] and [4].
Data source location	All data are from the Sierra Nevada Massif, SE Spain (Fig 1). Overall, the region falls within a rectangle defined by the Universal Transverse Mercator (UTM) coordinate system (30S) most of which are ≥ 2700 m a.s.l. (446,789 mE, 4,122,775 mN) - (531,182 mE, 4,122,775 mN) (531,182 mE, 4,083,836 mN) - (446,789 mE, 4,083,836 mN) The situational data were obtained in the field using a portable GPS (Garmin, eTrex 10) and later refined using “Google Earth” [1] and “Iberpix” (IGN) [2] visors.
Data accessibility	Physical Features of Waterbodies from Sierra Nevada, Spain. Mendeley Data V3, https://doi.org/10.17632/m9wzjwhffk.1
Related research article	[3] J.L. Diaz-Hernandez and A.J. Herrera-Martinez, Hydrological Characteristics and Paradoxes of Mediterranean High-mountain Water-bodies of the Sierra Nevada, SE Spain. Hydrology 6(3) (2019) 59. https://doi.org/10.3390/hydrology6030059

## Value of the Data

• This dataset represents a contribution to filling the present substantial void of information [8] on Mediterranean mountains. Its transboundary limits cover more than 10^7^ km^2^, and affect three continents [9].
• These areas are especially sensitive to climate change [10,11] and affect many aspects of nature, not only concerning the physical medium (mainly hydrology, hydrogeology, glaciology, meteorology, geomorphology and soil science), but also aspects of the biota (botanics, entomology, microbiology, aquatic and terrestrial ecology, among others).
• The data presented here define the present hydrological state of a Mediterranean massif. They make up the most influential component of the natural environment and the most easily affected by climate change. They are presented as reference points in a state of evolution when further delay is irresponsible.
• These data contribute to the correct use of these, or similar, habitats by providing understanding of the factors intervening in their management [12] and leaving aside assumptions or intuition, although they open up new questions. These data can shed light, for example, on the current debate on water management, or they can show phenomena of unknown scope, such as the different water behaviour of the two main watersheds.
• This dataset can also be used to research the relations between the hydrological cycle and associated local precipitation, or to study the biogeochemical cycles in habitats like this [13].
• Sierra Nevada is at present a Biosphere Reserve and is considered one of the 25 critical points of biodiversity [5], [6], [7]. Therefore, these data facilitate understanding of the behaviour of the different species [14], many of them endemic, in the different moisture conditions of the area.

## 1 Data Description

**Fig. 1:**
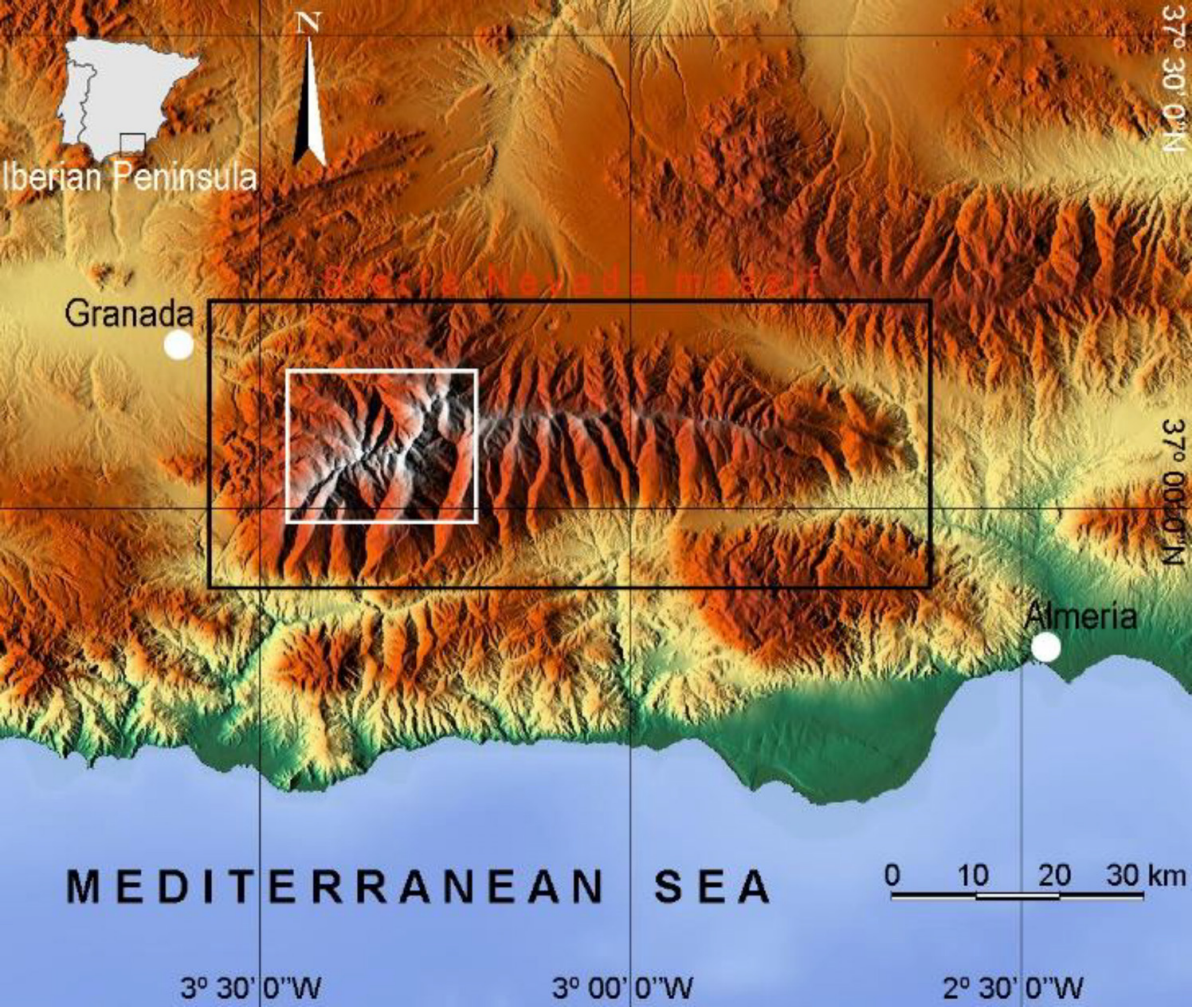
Situation of the Sierra Nevada Massif (black box). The smaller white box shows the nuclear area (Fig. 2) where the analysed water-bodies are located. Modified figure from its equivalent of [4].

The data presented correspond to an exhaustive inventory of aquatic biotopes, in which the sample obtained coincides with the population. This database is completely original and corresponds to the aquatic biotopes included in the nucleus of Sierra Nevada (Fig. 1). It consists of three groups of data: (1) Identifiers, (2) numerical data (3) qualitative data.

**Fig. 2:**
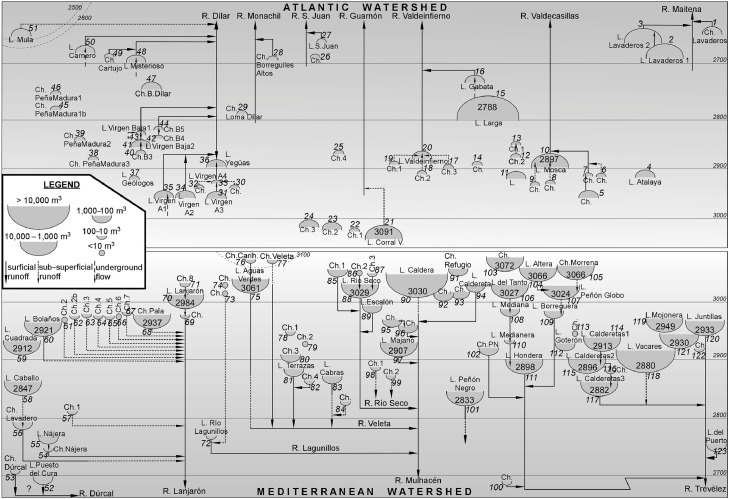
Positional relationship between the water-bodies of Sierra Nevada, ordered by watersheds and headwaters: the streams that drain them as surficial/sub-surficial runoff are indicated. The larger numbers included in the larger bodies are their heights (m a.s.l.). The small numbers near each water-body indicate the order, following clockwise from the upper right corner. Potential underground flows in the water-bodies (springs) and infiltrations occurring in the outflows are also shown. The water-body of Laguna Seca is not included because it is the lowest water-body (<2400 m a.s.l.). The water-bodies are enclosed in two boxes: the upper (Northern) box corresponds to the Atlantic watershed (Genil River), and the lower (Southern) box to the Mediterranean watershed (Guadalfeo River). Reprinted with permission from Hydrology [3].

The first column of the table identifies some common everyday denominations of the water-bodies, but these are few and many are subject of debate. The identifying data (UTM coordinates, 3rd and 4th columns) were obtained using a portable GPS (Garmin eTrex 10) and “Google Earth” and “Iberpix” (IGN) visors [1,2] (in lab) and determine unambiguously the position of the centre of each water-body. All are located on the 30S grid zone. Altitudes (5th column) were obtained from cartography by the National Geographical Institute (IGN) using the “Iberpix” [2] online visor, which was considered the most accurate. Fig. 2 gives a general outline of the relative positions of the 123 inventoried water-bodies, where the order number (2nd column) corresponds to that of the database. This ranking follows geographical criteria, beginning with the bodies found on the Atlantic watershed (upper northeastern angle) and continuing in an anti-clockwise direction.

**Fig. 3:**
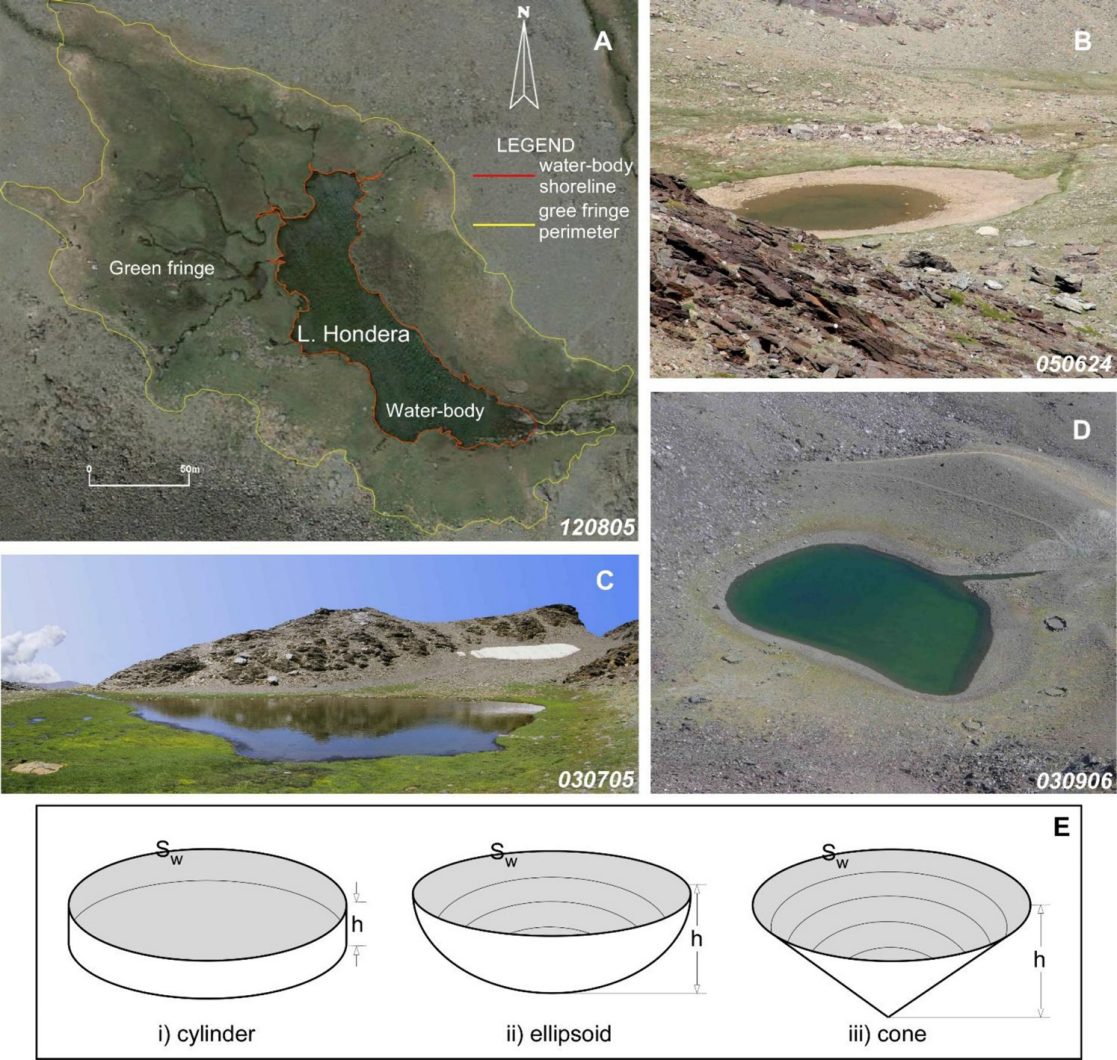
Some examples of water-bodies. (A) Aerial orthophotography of Laguna Hondera showing the main cartographic elements of this water-body (n^ber^ 111) and the associated green fringe. (B) The case of Laguna de Majano (n^ber^ 97), a water-body with 0.80 m depth, photographed during the dry spell. (C) Laguna de la Virgen (n^ber^ 34), a frequent type of water-body with 0.30 m depth. (D) Laguna del Caballo (n^ber^ 58) is a representative case of a deep water-body (5.4 m), with associated sparse narrow green fringe. (E) Set of three selected geometrical bodies (cylinder, ellipsoid and cone) whose volumetric formulas were used to estimate the water volumes of the natural water-bodies of Sierra Nevada (see text). S_w_ is the “equivalent surface” (see text). The number of each water-body (n^ber^) is the order number referred both in the Fig. 2 and in the dataset Table. The numbers in lower right corners specify the date of each photograph (year, month, day).

The numerical data evaluate the dimensions of each water-body – the length and width (6th and 7th columns) correspond to the dimensions of the rectangle in which each water-body was inscribed (in the field). There is [4] a close relationship between the water surface area obtained from cartography (8th column) and that of the circumscribed rectangle (R^2^=0.958, *p* < 0.0001), although it is obvious that the latter is larger. These data serve to calibrate the software used in the cartography (Canvas X Pro) using orthogonal aerial photographs. In this way, the shoreline (or length of the water border, 9th column), the surface area and perimeter of the green fringes (12th and 13th columns) were determined (Fig. 3A–D). Some values “0.0” in the two columns of green fringes indicate the absence of this vegetal cover.

The water depth (10th column) was measured in field with a metric tape at the deepest point, and water volumes (11th column) were obtained following the methodology stated below. Qualitative classifications are included in columns 14th, 15th and 16th.

## 2 Experimental Design, Materials and Methods

The environment is mountainous, poorly understood, and recognition was based on geomorphological evidence (depressions flooded by meltwater), sedimentological evidence (presence of clearly identifiable bottom sediments), water-level marks on rocky edges, and the presence of associated green fringes, taking into account all water-bodies of no matter what significance. Most of the water-bodies are distributed at altitudes above 2700 m a.s.l. Proof of the interest in conserving these water-bodies is the fact that the nucleus of Sierra Nevada was declared a Biosphere Reserve in 1986 [4] and it is one of the 25 biodiversity hotspots [5,6].

The circumstance that most of these water-bodies are ephemeral after the thaw (June-September inclusive), meant that field trips had to be carried out early in the summer months, as long as access was possible.

Simple measuring methods were used, backed up with cartographic software. Preliminary location was carried out in some cases using GPS.

The volume of water in each body was estimated using three geometrical figures whose base is the water surface area. For greater precision the “equivalent surface” (S_w_, Fig. 3E), was used: it represents the surface of each water-body and was obtained by cartography (8th column). The morphologies of the water-bodies were classified [3,4] according to three categories of the depth parameter h (Fig. 3E):

**Table:** 

(i)	*h*< 0.5m	*V_water body i)_ = V_cylinder_ = S_w_ × h*
(ii)	0.5< *h*< 2m	V_water body ii)_ = 1/2 × [V_ellipsoid_] = 1/2 × [4/3 × (S_w_ × *h)*]
(iii)	*h*> 2 m	V_water body iii)_ = V_cone_ = 1/3 × (S_w_ × h*)*

These volumes were compared [4] with the few existing bathymetrical data and show good correlation (R^2^=0.975, *p* < 0.0001).

Finally, the qualitative data [4] describe the patterns of the preponderant geological processes involved in the formation of the hollows where the water-bodies formed. The main formation processes were [4]: glacial (mainly frequent in the high peaks), and landsliding (below the previous ones, although may overlap each other). Additionally, we have considered another more heterogeneous category (“Others”). Each of these categories is subdivided into two others, according to their specific characteristics. The following abbreviations are used to designate these patterns in the Table:

**Table:** 

Glaciar:		
	Overdeepening	Ovrdp.
	Moraine dams	Mor. dam.
Landsliding:		
	Debris Flow	Deb-flow
	Rock-fall	Rock-fall
Others:		
	Anthropogenic	Anthr.
	No defined	N.D.

## Ethics Statements

Protected species were at no time put at risk by the work involved in this research.

## CRediT authorship contribution statement

**J.L. Diaz-Hernandez:** Conceptualization, Methodology, Formal analysis, Investigation, Resources, Writing – original draft, Writing – review & editing. **A.J. Herrera-Martinez:** Conceptualization, Methodology, Formal analysis, Investigation, Resources, Writing – original draft, Writing – review & editing.

## Declaration of Competing Interest

The authors declare that they have no known competing financial interests or personal relationships that could have appeared to influence the work reported in this paper.

## Appendix Supplementary materials

Image, application 1
